# Successful Multi-chamber Catheter Ablation of Persistent Atrial Fibrillation in Cor Triatriatum Sinister

**Published:** 2011-03-25

**Authors:** Andrew Gavin, Cameron B Singleton, Andrew D McGavigan

**Affiliations:** 1 Department of Cardiovascular Medicine, Flinders Medical Centre; 2 Faculty of Medicine, Flinders University of South Australia, South Australia 5042, Australia

**Keywords:** Cor Triatriatum Sinister, Atrial Fibrillation, Multi-chamber Catheter Ablation

## Introduction

Cor triatriatum sinister is a rare congenital cardiac anomaly with an incidence of between 0.1 and 0.4% [1,2] whereby the left atrium is functionally and anatomically divided into two separate chambers by a thin membrane. Although there are a limited number of reports of successful ablation of left atrial focal tachycardia and simple pulmonary vein isolation in individuals with cor triatriatum sinister [3-5], these involved ablation within a single atrial chamber only. To the best of our knowledge ablation in all three atrial chambers has not previously been described. We report a case of extensive ablation of long-lasting persistent atrial fibrillation (AF) encompassing pulmonary vein antral ablation with isolation combined with additional substrate modification involving ablation within both the supero-posterior and infero-anterior left atrial chambers in addition to the right atrium and the coronary sinus - a four chambered ablation approach.

## Case report

**Figure 1:**
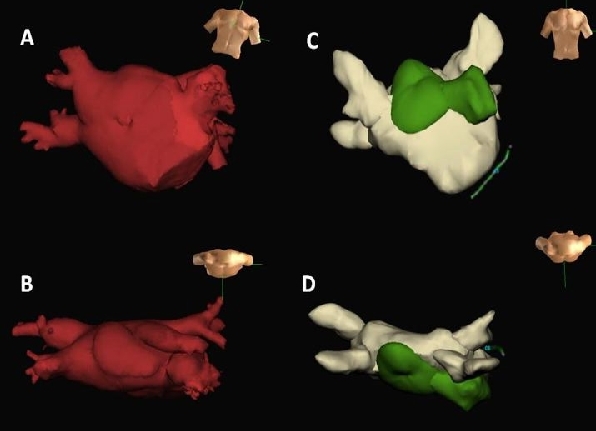
CT of left atrium in antero-posterior view (A) and viewed from above (B). Note the bulging anterior to the left sided veins which represents the anterior chamber. Panels C and D show the Ensite NavX geometry viewed in the same orientations. The infero-anterior chamber is shown in green and contains the left atrial appendage. The pulmonary veins drain into the supero-posterior chamber.

A 64 year old man with a 4 year history of long-lasting persistent drug refractory AF and previous failed attempts at cardioversion was referred for catheter ablation. There was no history of hypertension, coronary artery disease or obstructive sleep apnea. Previous transthoracic echocardiogram had been reported as demonstrating mildly dilated left atrium at 4.2cm with a membrane within the left atrium. There was mild mitral regurgitation with no evidence of impaired trans-mitral flow. The left ventricle was undilated (left ventricular end diastolic diameter 4.9cm) with normal systolic function (ejection fraction 70%). These findings were confirmed by a pre-operative multi-slice CT scan which clearly demonstrated a membrane dividing the left atrium into 2 chambers. The supero-posterior chamber received the 4 pulmonary veins whilst the infero-anterior chamber contained the left atrial appendage and was in direct continuity with the mitral valve (Figure 1a and 1b).

The patient was in AF at the commencement of the procedure. Under general anaesthesia, bilateral femoral venous access was obtained. A 6Fr quadrapolar catheter was advanced from the left femoral vein to the His position and a 7Fr steerable decapolar catheter was advanced from the right femoral vein to the coronary sinus. Intra-operative transesophageal echocardiogram (TEE) was performed to further delineate left atrial anatomy and aid trans-septal access to the left atrium.

**Figure 2:**
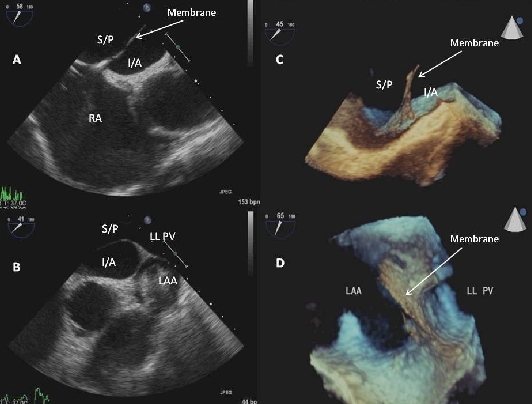
Standard transesophageal pictures showing septal insertion of membrane posterior to fossa (A) and lateral insertion at venous-appendage ridge (B). Panels C and D show insertion points on 3-Dimensional TEE imaging. Panel C shows fenestration of membrane and D demonstrates anatomical separation of appendage (LAA) and left lower pulmonary vein (LLPV) by membrane extending into left atrium. Abbreviations: S/P = Supero-Posterior left atrial chamber; I/A = Infero-Anterior left atrial chamber; LAA = Left Atrial Appendage; LLPV = Left Lower Pulmonary Vein.

Standard and 3-dimensional TEE confirmed the diagnosis of cor triatriatum with a fenestrated membrane arising from the anterior roof of the left atrium extending partly down the chamber in the antero-inferior direction (Figure 2 ). The septal insertion was just posterior to the fossa ovalis with the lateral insertion point being the appendage-venous ridge. A patent foramen ovale (PFO) was also shown.

Access to the infero-anterior chamber was gained via the PFO using an SL1 sheath advanced from the right femoral vein. A decapolar circular catheter (Reflexion Spiral X, St Jude Medical, St Paul, MN, USA) was advanced into this chamber via the sheath and a 4mm irrigated catheter (CoolPath Duo, St Jude Medical, St Paul, MN, USA) was passed alongside using a single wire, dual access approach. Intravenous heparin was used to maintain an ACT of 300-350 seconds. Both catheters were inserted into the antero-inferior chamber. The supero-posterior chamber was accessed relatively easily by rotating the catheters posteriorly under the membrane

A 3 dimensional multichamber geometry of the left atrium and pulmonary veins was created using the Ensite NavX system (St Jude Medical, St Paul, MN, USA) without CT integration. Given that the left atrium was functionally and anatomically divided by the membrane, the supero-posterior and infero-anterior aspects were created as separate chambers (Figure 1c and 1d). As per normal protocol at our centre a nasogastric tube was inserted to the gasto-esophageal junction and IV contrast was used to ascertain the esophageal position which was determined to be midline.

**Figure 3:**
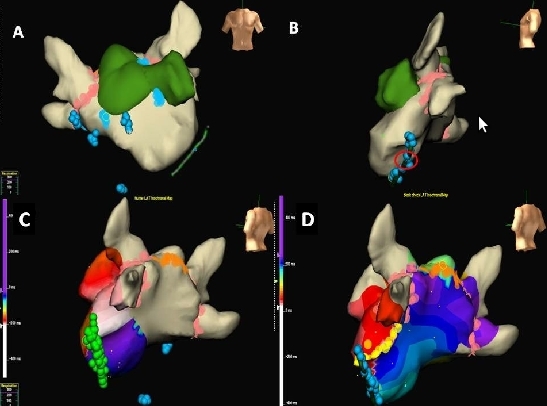
Panels A and B demonstrate lesion sets displayed on NavX geometry in the antero-posterior and left lateral projections. Lesions encircling the pulmonary veins are displayed as pink dots and ablation at sites of fractionation as blue dots. Sites of fractionation were targeted in the infero-anterior chamber at the base of the left atrial appendage, at the left side of the inter-atrial septum and in the right atrium at the high and low crista terminalis and the inter-atrial septum. Ablation was also performed within the coronary sinus (Panel B, blue dots) producing conversion to atrial tachycardia while ablating at highlighted area (red circle). Panel C demonstrates atrial tachycardia with a cycle length of 264ms. Coronary sinus activation is proximal to distal. Panel D shows the linear lesion sets (roof line in orange and mitral line in yellow) and activation pattern during pacing from the left atrial appendage. Block across the left atrial roof and mitral isthmus is demonstrated with earliest activation (green) anterior to the roof line adjacent to the site of pacing with latest activation (dark blue and purple) just posterior to the line. The absolute difference in timing is 150ms either side of roof line with caudo-cranial activation of the posterior LA all of which indicate successful line of block. Similarly, relatively early signals (red) are seen anterior to the mitral isthmus line with late signals (blue) immediately posterior to the line with the inferior portion of the posterior wall of the LA being activated in a septal – lateral fashion indicating block across the mitral isthmus.

**Figure 4:**
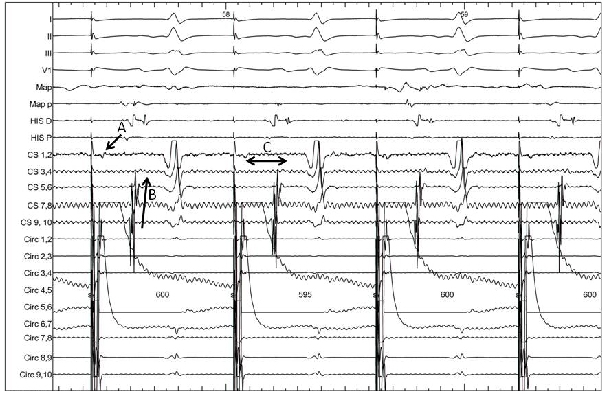
EGM demonstrating block across the mitral line. With the circular catheter in the left atrial appendage anterior to the mitral line an early signal is seen in CS 1,2 (A) which is lateral to the line of block. The coronary sinus is activated from proximal to distal (B) with a 220ms delay between CS 1,2 and CS 3,4 (C) indicating block across line.

The strategy employed was pulmonary venous antral ablation with isolation as the end point. Further ablation within the supero-posterior chamber was performed with linear ablation across the roof connecting the right and left superior pulmonary veins. Complex fractionated electrograms were then targeted within the two left atrial chambers, the right atrium and within the coronary sinus (Figure 3a and 3b). During ablation within the distal coronary sinus, AF organized to atrial tachycardia with a cycle length of 264ms. Activation mapping confirmed perimitral flutter (Figure 3c). Linear ablation connecting the left inferior vein to the mitral annulus produced termination of tachycardia with return to sinus rhythm. Further ablation within the distal coronary sinus at the epicardial aspect of the mitral line was required to produce block (Figure 4). At the end of the procedure all 4 veins remained isolated with block across the left atrial roof and mitral isthmus as assessed by activation mapping whilst pacing from the left atrial appendage (Figure 3d and Figure 4) and by demonstrating double potentials along the lines.

During 7 months of follow up the patient has remained symptomatically free of atrial fibrillation without any antiarrhythmic drugs.  24 hour holter monitoring 3 and 6 months post procedure did not show any evidence of atrial fibrillation.

## Discussion

Cor triatriatum sinister is commonly thought to result from failure of the common pulmonary vein to become fully incorporated into the left atrium during foetal development. This results in a fibro muscular septum like structure which divides the left atrium into a postero-superior chamber receiving the pulmonary veins and an antero-inferior chamber in direct continuity with the mitral valve. It usually presents in childhood, often in association with other cardiac abnormalities. However with improvements in diagnostic imaging it is being diagnosed with increasing frequency in adults [6] either incidentally or when they present with atrial fibrillation or clinical signs of mitral stenosis [7,8].  Distortion to muscle fiber architecture and electrical remodeling in response to altered left atrial pressure and volume may also predispose to atrial arrhythmias. This, coupled with increasing frequency of diagnosis and the rapidly expanding availability of catheter ablation for the treatment of atrial fibrillation, makes it inevitable that an increasing number of these patients will be seen.

Although there are two previous case reports of AF ablation in the literature, these involved only single chamber ablation with isolation of the pulmonary veins and no attempt at additional substrate modification. It is generally recognized that pulmonary vein ablation with isolation alone is insufficient in the treatment of persistent atrial fibrillation and that additional substrate modification is required [9-12]. To the best of our knowledge there have been no other reports of extensive multi-chamber ablation in a patient with cor triatriatum and long-lasting persistent atrial fibrillation. In this case, successful substrate modification by means of linear ablation and ablation of complex fractionated electrograms was performed in both left atrial chambers in addition to ablation within the coronary sinus and right atrium - a four chamber ablation in addition to simple pulmonary vein antral ablation.

The use of a 3 dimensional mapping system and good pre- and intra-operative imaging were essential in the planning and execution of the ablation procedure. Our experience would suggest that these patients can be effectively treated with a good outcome.
